# Changes in Respiratory Parameters and Fin-Swimming Performance Following a 16-Week Training Period with Intermittent Breath Holding

**DOI:** 10.1515/hukin-2015-0111

**Published:** 2015-12-30

**Authors:** Vasileios Stavrou, Argyris G. Toubekis, Eleni Karetsi

**Affiliations:** 1 Department of Physical Education and Sport Science, University of Thessaly, Trikala, Greece; 2 Faculty of Physical Education and Sport Science, Department of Aquatic Sports, University of Athens, Athens, Greece; 3 Medical School, Department of Cardio-Pulmonary Testing, Pulmonary Clinic, University of Thessaly, Larissa, Greece

**Keywords:** hypoventilation, oxygen saturation, youth fin-swimmers

## Abstract

The purpose of this study was to examine the effects of training with intermittent breath holding (IBH) on respiratory parameters, arterial oxygen saturation (SpO2) and performance. Twenty-eight fin-swimming athletes were randomly divided into two groups and followed the same training for 16 weeks. About 40% of the distance of each session was performed with self-selected breathing frequency (SBF group) or IBH (IBH group). Performance time of 50 and 400 m at maximum intensity was recorded and forced expired volume in 1 s (FEV1), forced vital capacity (FVC), peak expiratory flow (PEF) and SpO2 were measured before and after the 50 m test at baseline and post-training. Post-training, the respiratory parameters were increased in the IBH but remained unchanged in the SBF group (FEV1: 17 ±15% vs. −1 ±11%; FVC: 22 ±13% vs. 1 ±10%; PEF: 9 ±14% vs. −4 ±15%; p<0.05). Pre compared to post-training SpO2 was unchanged at baseline and decreased post-training following the 50 m test in both groups (p<0.05). The reduction was higher in the IBH compared to the SBF group (p<0.05). Performance in the 50 and 400 m tests improved in both groups, however, the improvement was greater in the IBH compared to the SBF group in both 50 and 400 m tests (p<0.05). The use of IBH is likely to enhance the load on the respiratory muscles, thus, contributing to improvement of the respiratory parameters. Decreased SpO2 after IBH is likely due to adaptation to hypoventilation. IBH favours performance improvement at 50 and 400 m fin-swimming.

## Introduction

Fin-swimming athletes often use reduced breathing frequency (RBF) in their training sessions to simulate competitive conditions. Exercise with RBF causes hypoventilation and leads to reduced oxygen saturation while increasing the heart rate (HR) and cardiac output (Woorons et al., 2011). Some of the reported changes during exercise with RBF (i.e. hypoventilation) are similar to those that have already been reported after adequate duration and submaximal intensity of intermittent hypoxic training (IHT) (Czuba et al., 2011; Dufour et al., 2006). In this case the RBF practice may be an alternative of hypoxic training, although hypercapnia may also occur under these exercise conditions (Woorons et al., 2010). Furthermore, during fin-swimming training, periods of apnea may be used to cover short distances underwater. This practice may be characterised as dynamic apnea and it may also induce specific responses (i.e. combination of hypoxia and hypercapnia) (deBruijn et al., 2008). It is suggested that apnea training may be beneficial for performance in sports (Lemaitre et al., 2010). However, static apnea as well as training with dynamic apnea does not improve short or middle distance performance in synchronised swimmers and swimmers (Chatard et al., 1999; Lemaître et al., 2009). In addition to enhanced adaptive response after IHT or training with RBF (Dufour et al., 2006; Woorons et al., 2008; Czuba et al., 2011), a simultaneous increase in expiratory parameters such as the forced expiratory volume in 1 s (FEV1), and forced vital capacity (FVC) in aquatic sports have been reported after training with apnea (Lemaître et al., 2009). It would be interesting if the aforementioned effects could improve performance after a training period with RBF or apnea. In fact, a 4 week training period with RBF showed a reduced level of acidosis in the blood at exercise intensity of 90% of the maximum heart rate (Woorons et al., 2008). Such an adaptation is beneficial in sports that are conducted in the aquatic environment and require intermittent breath holdings or short duration apneas in training or competition (fin-swimming, synchronised swimming). Despite its promising cardiac and metabolic effect, this type of training has not been evaluated with maximum intensity efforts that simulate competitive conditions. However, it is impossible to control the breath holdings and apneas or the level of expiration during training in the aquatic environment. Such a control requires specific equipment for continuous evaluation of respiratory parameters (Woorons et al., 2014). Because of the respiratory control constrains, experimental training could be performed with RBF and/or short duration apneas, however, these respiratory actions are not easy to distinguish in the real training setting (i.e. the fin-swimmer may, or may not expire during a given distance). In this regard, it is not possible to separate the effects of RBF with those of apnea.

The purpose of this study was to investigate the effect of training with intermittent breath holdings (IBH), which may include periods of RBF and apnea, on respiratory parameters, arterial oxygen saturation and performance in a 50 m apnea and 400 m surface competitive fin-swimming, and to compare it with the same training conducted with self-selected breathing frequency (SBF). We hypothesized that IBH training would be more beneficial for fin-swimming performance since it combines characteristics and adaptations induced by RBF and apnea training.

## Material and Methods

### Participants

Twenty-eight fin-swimming youth athletes with 3.5 ±1.2 years of competitive experience at the national level participated in the study. They were randomly divided into two groups i.e. the intermittent breath holding group (IBH) (n=14, age: 15.8 ±1.0 years, body height: 167 ±8 cm, body mass: 59.1 ±9.9 kg, body fat: 11.8 ±2.7%) and the self-selected breathing frequency group (SBF) (n=14, age: 15.4 ±1.4 years, body height: 167 ±9 cm, body mass: 56.7 ±8.1 kg, body fat: 10.6 ±2.5%). Seven males and seven females were included in each group. The study was approved by the Ethical Committee of the University of Thessaly and was conducted according to the declaration of Helsinki. All participants and one of their parents provided written consent to participate in the study which was conducted during the specific preparatory training period (November to February).

### Measures

The effects of a 16-week training period were examined in two groups of fin-swimming athletes. Both groups followed the same training content in all sessions. Athletes participating in the experimental group applied IBH at 40% of each training session while athletes in the control group breathed at their self selected breathing frequency (SBF group). The study design included repeated measurements (pre – post) in both groups (IBH and SBF) having as an independent variable the type of training and as dependent variables the performance (50 m apnea and 400 m surface fin-swimming), respiratory parameters, forced expiratory volume in 1 s (FEV_1_), forced vital capacity (FVC), peak expiratory flow (PEF), arterial oxygen saturation (SpO_2_) and heart rate (HR). All parameters were measured before and immediately after the 50 m apnea swimming at baseline (the beginning) and post-training, 16 weeks later (the end of the training period), applying the same procedures.

### Procedures

The anthropometric characteristics and the stage of biological maturation (Tanner and Whitehouse, 1976) were recorded on the first visit to the laboratory. Forty-eight hours later the tests were performed in the swimming pool over distances of 50 m apnea and 400 m surface fin-swimming after a controlled warm-up of about 1000 m. The equipment used by the athletes for testing the 50 m apnea and 400 m surface was the same in each testing session and in accordance with the international regulations for the fin-swimming competition (CMAS, 2010). Time was recorded with a digital handheld stopwatch (Cei-Ultrak 499, Cardena, California, USA). A portable gas analyzer (Micro Medical Plus, Medisave, UK) was used to measure FEV_1_, FVC and PEF. The measurements were performed according to the instructions of the ATS/ERS (Miller et al., 2005). Following a deep breath, the measurement of respiratory parameters was applied once. No repeated measurements were applied to avoid any effect of recovery duration. SpO_2_ was measured by pulse oximetry (Ri-fox, Riester, Germany) while simultaneously recording the HR. The hand and the finger were carefully dried before applying the oximeter’s probe within 10–12 s.

The training program lasted 16 weeks and each athlete took part in 5–6 training sessions per week. Duration of each training session was about 120 min and all sessions were conducted in a 50 m indoor swimming pool with water temperature of 26 ±1°C and environmental temperature of 23 ±1°C. Each training session included a warm-up (~5% of total session distance), a preparatory set (~20% of total session distance), the first main set of repetitions (~40% of total session distance), the second main set of training (~30% of total session distance) and a recovery set (~5% of total session distance). In the first main set of training, the athletes performed the repetitions divided in the IBH and SBF groups. The athletes of the IBH group during the main set (40% of the total training distance) did not use a respiratory snorkel while swimming with intermittent breath holdings and were divided into subgroups in order to better control the breathing restriction. The control group performed the same set with self-selected breathing frequency. The main sets of training with IBH were applied the same way throughout the intervention period and consisted of:

- Two sets of ten 50 m repetitions [2 × 10 × 50 m, (i.e. 2 × 10 × 27–32 s)] swimming apnea at 80% of the best individual performance with an interval of 15 s between repetitions and 3 min between sets.
- Fifteen repetitions of 100 m [15 × 100 m, (i.e. 15 × 65–74 s)] at 70% of the 100 m best performance with an interval of 10 s between repetitions and breathing every 25 m.
- Twenty repetitions of 25 m [20 × 25 m, (i.e. 20 × 12–13 s)] swimming apnea at intensity 80–90% of the best individual performance and starting each repetition every 18 s

Swimmers in the SBF were allowed to breathe at their own choice in all training sessions while using a snorkel. The pace of the main set was appropriately adjusted after a competition or after scheduled testing during training sessions (every 25–30 days).

### Statistical analysis

Analysis of variance for repeated measurements on three factors (2 repetitions × 2 measurements × 2 groups) was used to test the differences in dependent variables (FEV_1_, FVC, PEF, SpO_2_ and HR). A Tukey post-hoc test was used to locate any differences between means. Analysis of covariance, using as a covariate the baseline 50 and 400 m performance time values and as a dependent variable the change scores was used for the statistical analysis of performance at 50 and 400 m. For all the statistical analyses the statistical package Statistica 10 Software Trial was applied (Stat-Soft Inc, Tulsa, USA). The level of significance was set at p<0.05. The data are presented as mean values and standard deviation (Mean ±SD).

## Results

**Figure 1:**
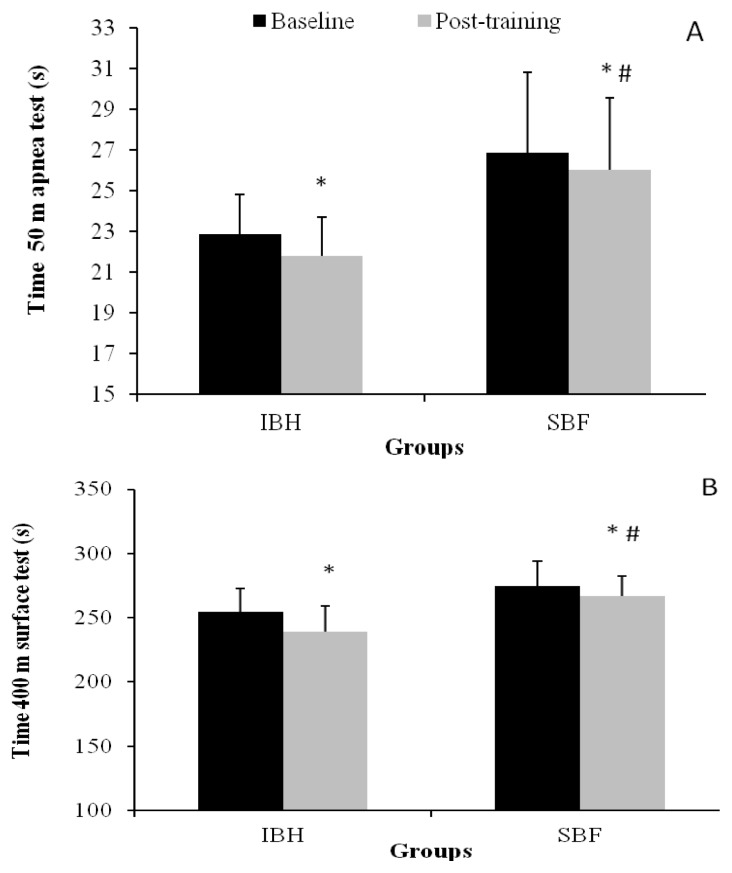
Changes in performance time of 50 m apnea (A) and 400 m surface fin-swimming tests (B) at baseline and after the intervention period in the intermittent breath holding (IBH) and self-selected breathing frequency (SBF) groups. *p<0.05 between baseline and post-training values, # p<0.05 between groups.

The post-training performance of the 50 m apnea and 400 m surface fin-swimming was improved in both groups compared with the baseline values (50 m: F1,26=39.0, 400 m: F1,26=48.7, Figure 1, p<0.01). Analysis of covariace was used to control for between groups baseline performance inequality in 50 and 400 m. A greater percentage and time improvement were observed in the IBH compared to SBF group in the 50 m test (4.7 ±3.5 vs. 3.1 ±3.0%, Figure 1; covariate adjusted percentage change: 5.1 ±3.3 vs. 2.6 ±3.3%; covariate adjusted time change: 1.29 ±0.86 vs. 0.53 ±0.86 s, F1,25=4.73, p<0.05). Similarly, the 400 m time improvement in the IBH was greater compared to the SBF group (6.0 ±3.9 vs. 2.8 ±2.7%, Figure 1; covariate adjusted percentage change: 6.4 ±3.5 vs. 2.4 ±3.5; covariate adjusted time change: 18.86 ±9.19 vs. 6.36 ±9.19 s, F1,25=8.02, p<0.01).

During baseline measurements no changes were observed before and after the 50 m apnea in both groups and for all measured respiratory parameters (Table 1, p>0.05). After the training period, FEV1 and FVC decreased at the end of the 50 m apnea compared to the starting values in the IBH, however, not in the SBF group (Table 1, p<0.05). PEF was unchanged before and after the 50 m test at baseline and post-training (Table 1, p>0.05). At baseline the respiratory parameters were similar between groups and were increased post-training in the IBH, but not in the SBF group (p<0.05). The post-training values were higher in the IBH compared to the SBF group (F1,26=7.95, Table 1, p<0.05).

**Figure 2:**
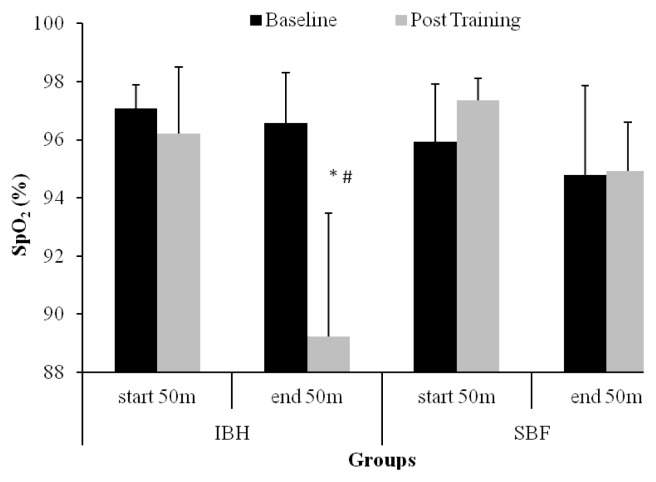
Changes in arterial oxygen saturation before and after the 50 m apnea test in the intermittent breath holding (IBH) and self-selected breathing frequency (SBF) groups. *p<0.05 between baseline and post-training values, # p<0.05 between groups.

In the baseline measurement, SpO2 at the end of the 50 m apnea remained unchanged compared to the values at the start of the effort in both groups (IBH: −1.0 ±2.0%, SBF: −1.0 ±3.0%, Figure 2, p>0.05). After the training period a significant reduction in SpO2 appeared in both groups and this reduction was greater in the IBH compared to the SBF group (−7.3 ±3.4 vs. −2.5 ±1.6%; F1,26=9.2, Figure 2, p<0.05).

The heart rate was not different between groups and showed no change after the training period. Furthermore, no interaction was observed between groups and measurements (F1,26=0.031, p>0.05). There was no difference between the baseline and post training nor between group values in anthropometric variables (p>0.05).

## Discussion

A 16-week training period with IBH or SBF was applied in fin-swimming athletes in the present study. The results of the study indicate that the IBH applied by the youth athletes seemed to positively affect the respiratory parameters associated with the power of exhalation and caused increased tolerance to hypoxia. The aforementioned factors are likely to help in achieving a better race performance. In the present study performance was improved after training with IBH and SBF at a 50 m apnea and at 400 m surface tests. However, the IBH showed greater improvement compared to the SBF group (5% and 6% vs. ~3%).

Previous studies have suggested that during exercise in a supine position in the water, hypoxia is not developing owing to a small difference of alveolar to arterial O2 content occurring by a better blood flow and O2 distribution in the working muscles (Woorons et al., 2008). Instead, hypercapnia was reported after RBF swimming (West et al., 2005). However, there are reports indicating that hypoxia may occur during maximal and submaximal intensity swimming (Miyasaka et al., 2002; Woorons et al., 2014). In land exercise, repeated apnea or expiration close to the residual volume may contribute to progressive reduction of O2 saturation and this may be also accompanied by hypercapnia (deBruijn et al., 2008; Woorons et al., 2007; Woorons et al., 2010). Nevertheless, combined effects of hypoxia and hypercapnia in addition to dynamic apneic conditions have to be considered when trying to explain the improved performance in the 400 m test in the present study. With the present experimental real-training condition setting, in the aquatic environment, it is impossible to separate the RBF from apnea training effects. Therefore, our findings should be interpreted according to physiological responses induced by both the above mentioned interventions.

Previous studies have reported that six weeks of training, supplemented with 24 to 40 min of exercise at hypoxic conditions, increased pH regulation and improved the mitochondria function while increasing the speed at the second ventilatory threshold and endurance performance (Dufour et al., 2006; Ponsot et al., 2006; Zoll et al., 2006). Such changes contribute significantly to improved performance in a ~4 min effort like in the present study. Furthermore, repeated apnea induces transient decrements in SaO2 and stimulates serum erythropoietin increases (deBruijn et al., 2008) which may contribute to enhanced O2 carrying capacity. Compared to only 4 hours of exercise in hypoxic conditions (Dufour et al., 2006; Ponsot et al., 2006) and only 15 maximum apneas (deBruijn et al., 2008) in previous studies, more than 70 hours of IBH training was applied during the 16 week period in the present study. Although hypoxic or hypercapnic conditions were not expected for the total duration of the 70 hours, since exercise was performed in normobaric conditions, it seems that this may be an adequate stimulus to induce adaptations similar to those observed with intermittent hypoxic training and apneas.

The 400 m test was completed in about 4 min and besides the high aerobic energy demand the anaerobic glycolysis was highly activated and contributed to more than 20 to 25% of the total energy demand (Gastin, 2001). In fin-swimming the propulsion of the athlete is mainly achieved through the power exerted in the monofin by large muscle groups of the lower limbs (CMAS, 2010). The systematic use of the lower limbs in fin-swimming is likely to increase concentration of lactate during training and this could be higher with the IBH compared to the SBF training mode. Higher lactate values were reported with RBF (Woorons et al., 2010) and the repeated and higher activation of glycolysis during RBF training may have led to increased buffering capacity (Woorons et al., 2008). However, it should be noted that there are studies suggesting that exercise with RBF does not affect lactate production (Town and Vanness, 1990; West et al., 2005).

Whatever the case, the potential of increased lactate production combined with increased hypercapnia during exercise is likely to cause increased metabolic and respiratory acidosis during training with IBH leading to increased buffering capacity. In the study of Woorons et al. (2008) despite increased buffering capacity, the exercise time to exhaustion was not improved after training with RBF. However, this study used a time limit test at 100% of VO2max to evaluate performance and was probably lower than the maximum intensity applied in the present study. A likely increase of buffering capacity and adaptations that enhance the aerobic potential after training with RBF was expected to have an impact on higher intensity efforts such as the 400 m maximum intensity test applied in the present study.

The 50 m test requires energy predominantly from anaerobic metabolism (Gastin, 2001) and any improvement in performance should be attributed to anaerobic power increment with training. Previous studies have shown that anaerobic capacity and swimming performance were increased after hypoxic training (Truijens et al., 2003; Ogita, 2006). Furthermore, coordination of swimming movements was improved after apnea training (Lemaitre et al., 2009) and this supports performance enhancement observed in the 50 m apnea fin-swimming test. Improved tolerance to hypoxemia observed in the IBH group following the 50 m test after the 16 weeks of training could have also contributed to a positive effect on performance.

During maximum intensity swimming the respiratory system is maximally taxed, since swimmers normally reach VO2max at the end of a 400 m effort (Rodriquez, 2000). Systematic swimming training improves lung function as evidenced by the functional residual capacity, total lung capacity and enhances expiratory flows such as FEV1 (Courteix et al., 1997). However, such long term changes have not been confirmed in adult endurance athletes (Kippelen et al., 2005), although, increased lung volumes have been reported in well-trained breath hold divers familiarized with apnea training (Overgaard et al., 2006). In the present study, duration of training was long and in combination with repeated apnea applied within each session, it may have induced an increase in the intra-thoracic pressure inducing positive effect on the respiratory muscle function. In agreement with our findings, Lemaître et al. (2009) reported about 2% improvement of FEV1 and PEF after apnea training of 12 weeks. As no control group was used in the last study, they could not determine whether the increased FEV1 was explained by swimming training alone or by both swimming and apnea training. In our study there was a control group, thus, the improvement in inspiratory muscles strength occurred owing to the type of training applied in the present research. Improvement in inspiratory muscle strength has a positive impact on performance during maximal exercise (Volianitis et al., 2001). This was evident in the IBH group and possibly contributed to enhanced 400 m performance compared to the SBF group.

Respiratory muscle training may improve the respiratory muscle function during submaximal exercise at 70% of VO2max, however, with no difference in SaO2 (Stuessi et al., 2001). A 7% decrease in SpO2 was observed in the IBH group at the end of the 50 m apnea following the training period. Similarly, decreased levels of SaO2 were reported after maximum intensity swimming (Miyasaka et al., 2002). A lower pH or a higher VO2max compared with the baseline values may have accentuated the decrease in SaO2. It has been reported that individuals with high VO2max or those who are able to sustain low pH during maximal exercise are more prone to develop exercise induced hypoxemia (Nielsen, 2003). A likely greater VO2max improvement in the IBH group may have caused a greater hypoxemia after the 50 m test. It is also likely that widening of the alveolar to arterial difference in O2 pressure or a decreased sensitivity to CO2 may have contributed to lower SaO2 levels (Dempsey and Wagner, 2006). Lemaître et al. (2009) showed a decreased end-tidal carbon dioxide pressure after 12 weeks of breath holding training, indicating a decreased stimulus for breathing, (possibly because of reduced central chemoreceptor sensitivity and drive to breath), which may lead to a greater hypoxemia. Whether this was an adaptation to IBH training should be examined in future studies. The reduced SpO2 after 50 m apnea may be due to adjustments that promote faster release of O2 so as to supply it to the muscles. This allows athletes to use larger proportions of the available O2 during intense exercise.

Training with IBH improved performance in 50 m apnea and 400 m surface fin-swimming tests compared to training with self-selected breathing. The long term application of IBH likely combines hypoxia-hypercapnia and the apnea stimulus, and seems to be advantageous for performance enhancement. Nevertheless, the haematological or metabolic factors involved in this improvement have not been examined in the present study. Improved performance in the group with IBH seems to be a combined effect of increased tolerance to hypercapnia and apnea during exercise and athlete’s adaptation in a way that facilitates exercise under hypoventilated-hypercapnic conditions and probably leads to increased metabolic adaptations (e.g. buffering capacity) compared to the group which undertook self-selected respiratory frequency.

Intermittent breath holding may be applied during 25, 50 and 100 m repetitions for fin-swimming training. This practice is probably useful to be applied systematically for a long duration period (i.e. 16 weeks), as part of a training session and at submaximal intensity (i.e. 70% of best performance). Improved performance is expected to be higher after intermittent breath holding compared to self selected breathing frequency in the 50 and 400 m distance fin-swimming competition. This improvement is possibly due to the combined effect of the hypoxic, hypercapnic or apneic stimulus applied in the present experimental setting.

## Figures and Tables

**Table 1:** Respiratory parameters and heart rate response before and after the 50 m test at baseline and post-training in the IBH and SBF groups. Percentage change is from baseline to post training average of before and after 50 m values (mean ± SD).

		Baseline		Post-Training		
		Before	After	Before	After	
	Group	50 m	50 m	50 m	50 m	% change
FEV_1_ (L) predicted (%)	IBH	3.3 ± 0.7	3.2 ± 0.7	4.1 ± 0.8*#	3.8 ± 0.8*†#	17 ± 15
		103.0±16.1	98.9±17.1	129.1±20.9	119.3±23.8	
	SBF	2.7 ± 0.5	2.9 ± 0.5	2.9 ± 0.4	2.7 ± 0.5†	−1 ± 11
		96.8±22.5	101.6±20.9	103.2±21.3	95.0±24.4	

FVC (L) predicted (%)	IBH	3.3 ± 0.7	3.1 ± 0.7	4.3 ± 0.7*#	3.9 ± 0.6*†#	22 ± 13
		85.7±14.4	81.2±14.4	112.5±15.1	102.0±10.6	
	SBF	2.7 ± 0.6	2.9 ± 0.5	2.9 ± 0.5	2.7 ± 0.5†	1 ± 10
		80.4±19.5	85.0±18.6	87.8±20.9	79.5±19.3	

PEF (L·s^−1^) predicted (%)	IBH	4.2 ± 0.8	4.4 ± 0.6	4.9 ± 1.0*	4.7 ± 0.8	9 ± 14
		97.5±15.3	103.5±12.8	114.9±21.1	110.1±18.0	
	SBF	3.8 ± 0.9	3.9 ± 0.9	3.8 ± 0.8	3.6 ± 0.7	−4 ± 15
		89.5±19.9	91.1±20.5	89.9±17.9	83.6±15.8	

HR (b·min^−1^)	IBH	90 ± 9	172 ± 16†	95 ± 12	176 ± 10†	4 ± 7
	SBF	104 ± 17	172 ± 16†	99 ± 9	170 ± 13†	−2 ± 9

IBH: Intermittent breath holding group, SBF: self-selected breathing frequency group, FEV_1_: forced expired volume in 1 s, FVC: forced vital capacity, PEF: peak expiratory flow, HR: heart rate.

* p<0.05 between baseline and post-training values.

# p<0.05 between groups.

† p<0.05 between pre and post the 50 m test.
